# Chemical graph generators

**DOI:** 10.1371/journal.pcbi.1008504

**Published:** 2021-01-05

**Authors:** Mehmet Aziz Yirik, Christoph Steinbeck

**Affiliations:** Friedrich Schiller Universität Jena, Institute for Inorganic and Analytical Chemistry, Jena, Germany; Museum für Naturkunde Berlin, GERMANY

## Abstract

Chemical graph generators are software packages to generate computer representations of chemical structures adhering to certain boundary conditions. Their development is a research topic of cheminformatics. Chemical graph generators are used in areas such as virtual library generation in drug design, in molecular design with specified properties, called inverse QSAR/QSPR, as well as in organic synthesis design, retrosynthesis or in systems for computer-assisted structure elucidation (CASE). CASE systems again have regained interest for the structure elucidation of unknowns in computational metabolomics, a current area of computational biology.

## History

**Fig 1:**
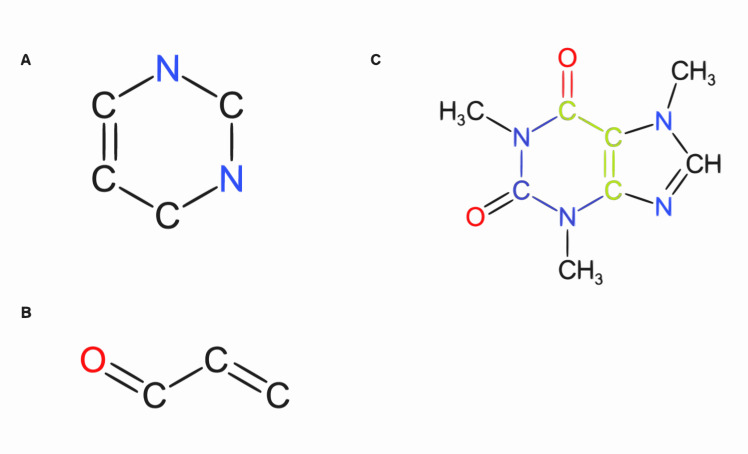
Overlapping substructures of caffeine. Two substructures of a caffeine molecule are given, **(A)** and **(B)**. The overlap of these substructures is highlighted in green in the caffeine structure **(C)**.

Molecular structure generation is a branch of graph) generation problems. Molecular structures are graphs with chemical constraints such as valences), bond multiplicity and fragments. These generators are the core of CASE systems. In a generator, the molecular formula is the basic input. If fragments are obtained from the experimental data, they can also be used as inputs to accelerate structure generation. The first structure generators were versions of graph generators modified for chemical purposes. One of the first structure generators was CONGEN,[1] originally developed for the DENDRAL project, the first artificial intelligence project in organic chemistry.[2] CONGEN dealt well with overlaps in substructures (Fig 1). The overlaps among substructures rather than atoms were used as the building blocks. For the case of stereoisomers, symmetry group calculations were performed for duplicate detection.

After DENDRAL, another mathematical method, MASS[3], a tool for mathematical synthesis and analysis of molecular structures, was reported. As with CONGEN, the MASS algorithm worked as an adjacency matrix generator. Many mathematical generators are descendants of efficient branch-and-bound methods from Igor Faradjev[4] and Ronald C. Read's orderly generation method.[5] Although their reports are from the 1970s, these studies are still the fundamental references for structure generators. In the orderly generation method, specific order-check functions are performed on graph representatives, such as vectors. For example, MOLGEN[6] performs a descending order check while filling rows of adjacency matrices. This descending order check is based on an input valence distribution. The literature classifies generators into two major types: structure assembly and structure reduction. The algorithmic complexity and the run time) are the criteria used for comparison.

## Structure assembly

The generation process starts with a set of atoms from the molecular formula. In structure assembly, atoms are combinatorically connected to consider all possible extensions. If substructures are obtained from the experimental data, the generation starts with these substructures. These substructures provide known bonds in the molecule. One of the earliest attempts was made by Hidetsugu Abe in 1975 using a pattern recognition-based structure generator.[7] The algorithm had two steps: first, the prediction of the substructure from low-resolution spectral data; second, the assembly of these substructures based on a set of construction rules. Hidetsugu Abe and the other contributors published the first paper on CHEMICS,[8] which is a CASE tool comprising several structure generation methods. The program relies on a predefined non-overlapping fragment library. CHEMICS generates different types of component sets ranked from primary to tertiary based on component complexity. The primary set contains atoms, i.e., C, N, O and S, with their hybridization. The secondary and tertiary component sets are built layer-by-layer starting with these primary components. These component sets are represented as vectors and are used as building blocks in the process.

Substantial contributions were made by Craig Shelley and Morton Munk, who published a large number of CASE papers in this field. The first of these papers reported a structure generator, ASSEMBLE.[9] The algorithm is considered one of the earliest assembly methods in the field. As the name indicates, the algorithm assembles substructures with overlaps to construct structures. ASSEMBLE overcomes overlapping by including a “neighbouring atom tag”. The generator is purely mathematical and does not involve the interpretation of any spectral data. Spectral data are used for structure scoring and substructure information. Based on the molecular formula, the generator forms bonds between pairs of atoms, and all the extensions are checked against the given constraints. If the process is considered as a tree), the first node of the tree is an atom set with substructures if any are provided by the spectral data. By extending the molecule with a bond, an intermediate structure is built. Each intermediate structure can be represented by a node in the generation tree. ASSEMBLE was developed with a user-friendly interface to facilitate use. The second version of ASSEMBLE was released in 2000.[10] Another assembly method is GENOA.[11] Compared to ASSEMBLE and many other generators, GENOA is a constructive substructure search-based algorithm, and it assembles different substructures by also considering the overlaps.

The efficiency and exhaustivity of generators are also related to the data structures. Unlike previous methods, AEGIS[12] was a list-processing generator. Compared to adjacency matrices, list data requires less memory. As no spectral data was interpreted in this system, the user needed to provide substructures as inputs. Structure generators can also vary based on the type of data used, such as HMBC, HSQC and other NMR data. LUCY is an open-source structure elucidation method based on the HMBC data of unknown molecules[13], and involves an exhaustive 2-step structure generation process where first all combinations of interpretations of HMBC signals are implemented in a connectivity matrix, which is then completed by a deterministic generator filling in missing bond information. This platform could generate structures with any arbitrary size of molecules; however, molecular formulas with more than 30 heavy atoms are too time consuming for practical applications. This limitation highlighted the need for a new CASE system. SENECA was developed to eliminate the shortcomings of LUCY.[14] To overcome the limitations of the exhaustive approach, SENECA was developed as a stochastic method to find optimal solutions. The systems comprise two stochastic methods: simulated annealing and genetic algorithms. First, a random structure is generated; then, its energy is calculated to evaluate the structure and its spectral properties. By transforming this structure into another structure, the process continues until the optimum energy is reached. In the generation, this transformation relies on equations based on Jean-Loup Faulon's rules.[15] LSD (Logic for Structure Determination)[16] is an important contribution from French scientists. The tool uses spectral data information such as HMBC and COSY data to generate all possible structures. LSD is an open source structure generator released under the General Public License (GPL). A well-known commercial CASE system, StrucEluc,[17] also features a NMR based generator. This tool is from ACD Labs and, notably, one of the developers of MASS, Mikhail Elyashberg. COCON[18] is another NMR based structure generator, relying on theoretical data sets for structure generation. Except J-HMBC and J-COSY, all NMR types can be used as inputs.

In 1994, Chinese scientists reported an integer partitioning)-based structure generator.[19] The decomposition of the molecular formula into fragments, components and segments was performed as an application of integer partitioning. These fragments were then used as building blocks in the structure generator. This structure generator was part of a CASE system, ESESOC.[20]

**Fig 2:**
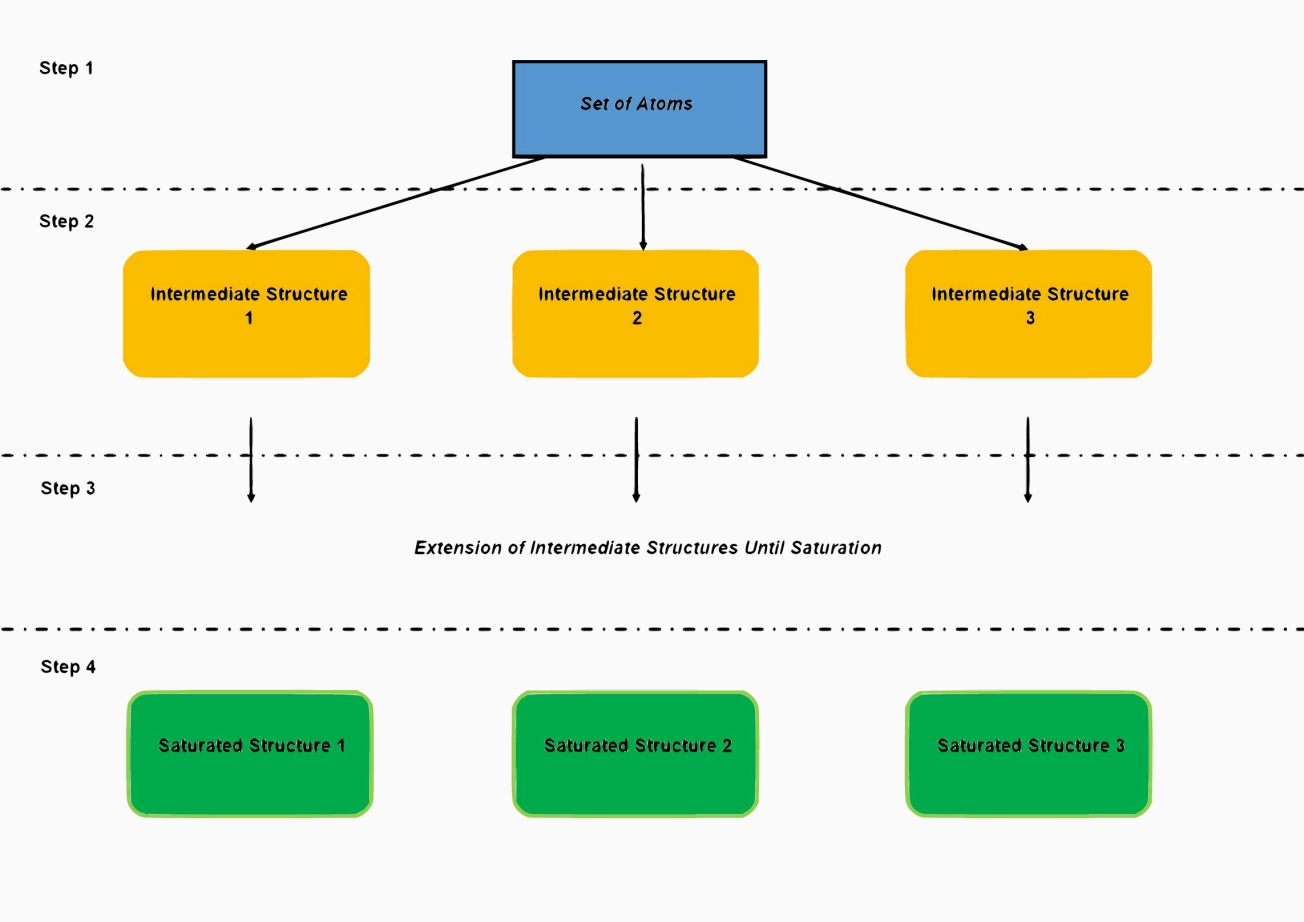
Breadth-first search generation. Molecular structure generation is explained step by step. Starting from a set of atoms, bonds are added between atom pairs until reaching saturated structures.

A series of stochastic generators was reported by Jean-Loup Faulon. The software, MOLSIG,[21] was integrated into this stochastic generator for canonical labelling and duplicate checks.[22] As for many other generators, the tree approach is the skeleton of Jean-Loup Faulon's structure generators. However, considering all possible extensions leads to a combinatorial explosion. Orderly generation is performed to cope with this exhaustivity. Many assembly algorithms, such as OMG,[23] MOLGEN and Jean-Loup Faulon's structure generator[24], are orderly generation methods. Jean-Loup Faulon's structure generator relies on equivalence classes over atoms. Atoms with the same interaction type and element are grouped in the same equivalence class. Rather than extending all atoms in a molecule, one atom from each class is connected with other atoms. Similar to the former generator, Julio Peironcely's structure generator, OMG, takes atoms and substructures as inputs and extends the structures using a breadth-first search method (Fig 2). This tree extension terminates when all the branches reach saturated structures.

OMG generates structures based on the canonical augmentation method from Brendan McKay's NAUTY package. The algorithm calculates canonical labelling and then extends structures by adding one bond. To keep the extension canonical, canonical bonds are added.[25] Although NAUTY is an efficient tool for graph canonical labelling, OMG is approximately 2000 times slower than MOLGEN.[26] The problem is the storage of all the intermediate structures. OMG has since been parallelized, and the developers released PMG (Parallel Molecule Generator).[27] MOLGEN outperforms PMG using only 1 core; however, PMG outperforms MOLGEN by increasing the number of cores to 10.

A constructive search algorithm is a branch-and-bound method, such as Igor Faradjev's algorithm, and an additional solution to memory problems. Branch-and-bound methods are matrix) generation algorithms. In contrast to previous methods, these methods build all the connectivity matrices without building intermediate structures. In these algorithms, canonicity criteria and isomorphism checks are based on automorphism groups from mathematical group theory. MASS, SMOG[28] and Ivan Bangov's algorithm[29] are good examples in the literature. MASS is a method of mathematical synthesis. First, it builds all incidence matrices for a given molecular formula. The atom valences are then used as the input for matrix generation. The matrices are generated by considering all the possible interactions among atoms with respect to the constraints and valences. The benefit of constructive search algorithms is their low memory usage. SMOG is a successor of MASS.

Unlike previous methods, MOLGEN is the only maintained efficient generic structure generator, developed as a closed-source platform by a group of mathematicians as an application of computational group theory. MOLGEN is an orderly generation method. Many different versions of MOLGEN have been developed, and they provide various functions. Based on the users' needs, different types of inputs can be used. For example, MOLGEN-MS[30] allows users to input mass spectrometry data of an unknown molecule. Compared to many other generators, MOLGEN approaches the problem from different angles. The key feature of MOLGEN is generating structures without building all the intermediate structures and without generating duplicates.

In the field, the recent studies are from Kimito Funatsu's research group. As a type of assembly method, building blocks, such as ring systems and atom fragments, are used in the structure generation.[31] Every intermediate structure is extended by adding building blocks in all possible ways. To reduce the number of duplicates, Brendan McKay's canonical path augmentation method is used. To overcome the combinatorial explosion in the generation, applicability domain and ring systems are detected based on inverse QSPR/QSAR analysis.[32] The applicability domain, or target area, is described based on given biological as well as pharmaceutical activity information from QSPR/QSAR.[33] In that study, monotonically changed descriptors (MCD) are used to describe applicability domains. For every extension in intermediate structures, the MCDs are updated. The usage of MCDs reduces the search space in the generation process. In the QSPR/QSAR based structure generation, there is the lack of synthesizability of the generated structures. Usage of retrosynthesis paths in the generation makes the generation process more efficient. For example, a well-known tool called RetroPath[34] is used for molecular structure enumeration and virtual screening based on the given reaction rules.[35] Its core algorithm is a breadth-first method, generating structures by applying reaction rules to each source compound. Structure generation and enumeration are performed based on Brendan McKay's canonical augmentation method. RetroPath 2.0 provides a variety of workflows such as isomer transformation, enumeration, QSAR and metabolomics.

Besides these mathematical structure generation methods, the implementations of neural networks, such as generative autoencoder models,[36, 37] are the novel directions of the field.

## Structure reduction

Unlike these assembly methods, reduction methods make all the bonds between atom pairs, generating a hypergraph. Then, the size of the graph is reduced with respect to the constraints. First, the existence of substructures in the hypergraph is checked. Unlike assembly methods, the generation tree starts with the hypergraph, and the structures decrease in size at each step. Bonds are deleted based on the substructures. If a substructure is no longer in the hypergraph, the substructure is removed from the constraints. Overlaps in the substructures were also considered due to the hypergraphs. The earliest reduction-based structure generator is COCOA,[38] an exhaustive and recursive) bond-removal method. Generated fragments are described as atom-centred fragments to optimize storage, comparable to circular fingerprints[39] and atom signatures[40]. Rather than storing structures, only the list of first neighbours of each atom is stored. The main disadvantage of reduction methods is the massive size of the hypergraphs. Indeed, for molecules with unknown structures, the size of the hyper structure becomes extremely large, resulting in a proportional increase in the run time.

The structure generator GEN[41] by Simona Bohanec combines two tasks: structure assembly and structure reduction. Like COCOA, the initial state of the problem is a hyper structure. Both assembly and reduction methods have advantages and disadvantages, and the GEN tool avoids these disadvantages in the generation step. In other words, structure reduction is efficient when structural constraints are provided, and structure assembly is faster without constraints. First, the useless connections are eliminated, and then the substructures are assembled to build structures. Thus, GEN copes with the constraints in a more efficient way by combining these methods. GEN removes the connections creating the forbidden structures, and then the connection matrices are filled based on substructure information. The method does not accept overlaps among substructures. Once the structure is built in the matrix representation, the saturated molecule is stored in the output list. The COCOA method was further improved and a new generator was built, HOUDINI.[42] It relies on two data structures: a square matrix of compounds representing all bonds in a hyper structure is constructed, and second, substructure representation is used to list atom-centred fragments. In the structure generation, HOUDINI maps all the atom-centred fragments onto the hyper structure.

## Mathematical basis

### Chemical graphs

**Fig 3:**
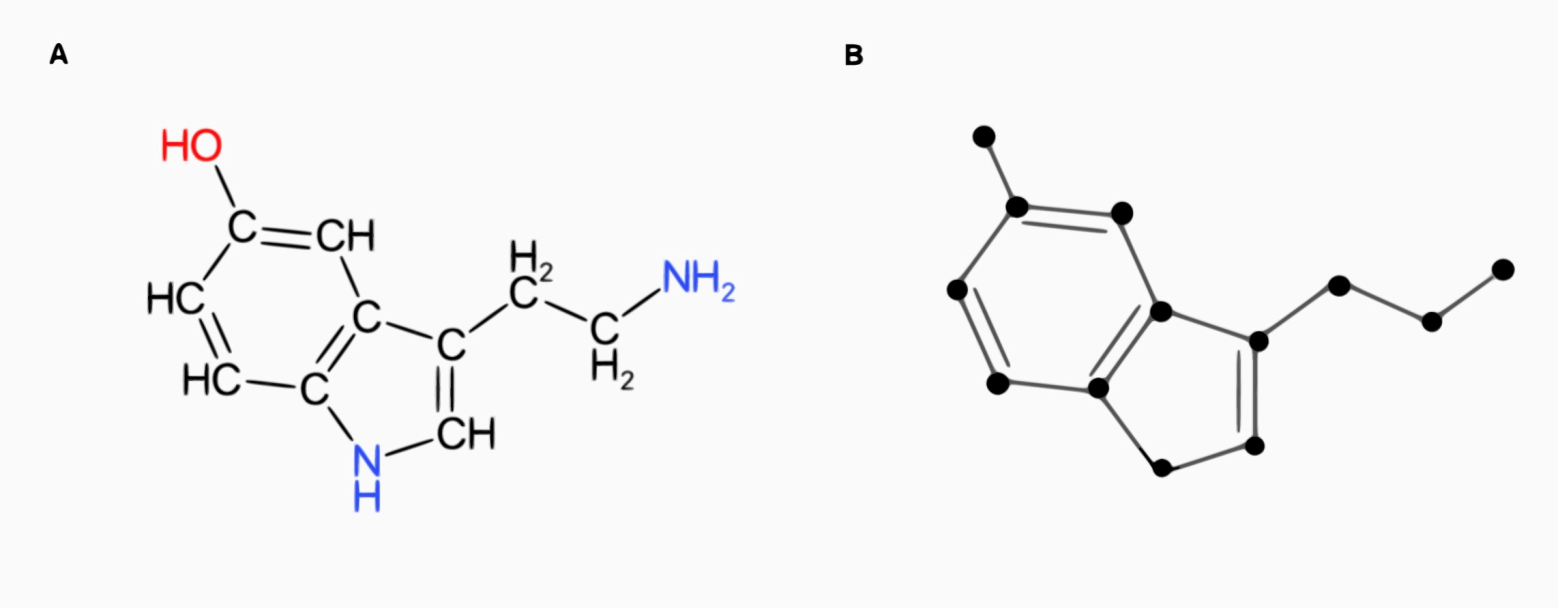
Graph representation of the serotonin molecule. **(A)** Molecular structure of serotonin. **(B)** Graph representation of the molecule.

In a graph representing a chemical structure, the vertices) and edges) represent atoms and bonds, respectively (Fig 3). The bond order corresponds to the edge multiplicity, and as a result, chemical graphs are vertex and edge-labelled graphs. A vertex and edge-labelled graph is described as a chemical graph where is the set of vertices, i.e., atoms, and is the set of edges, which represents the bonds.

In graph theory, the degree) of a vertex is its number of connections. In a chemical graph, the maximum degree of an atom is its valence), and the maximum number of bonds a chemical element can make. For example, carbon's valence is 4. In a chemical graph, an atom is saturated if it reaches its valence. A graph is connected) if there is at least one path between each pair of vertices. Although chemical mixtures[43] are one of the main interests of many chemists, due to the computational explosion, many structure generators output only connected chemical graphs. Thus, the connectivity check is one of the mandatory intermediate steps in structure generation because the aim is to generate fully saturated molecules. A molecule is saturated if all its atoms are saturated.

### Symmetry groups for molecular graphs

For a set of elements, a permutation is a rearrangement of these elements.[44] An example is given below (Table 1):

**Table 1:** Permutation of set of integers.

*x*	1	2	3	4	5	6	7	8	9	10	11
*f(x)*	4	2	11	6	1	5	8	9	7	10	3

The second line of Table 1 shows a permutation of the first line. The multiplication of permutations, *a* and *b*, a function composition, as shown below.

(a•b)(x)=a(b(x)) (1)

The combination of two permutations is also a permutation. A group, *G*, is a set of elements together with an associative binary operation • defined on *G* such that the following are true:

- There is an element *I* in *G* satisfying *g*•*I* = *g*, for all elements *g* of *G*.
- For each element of G, there is an element *g*^−1^ such that *g*•*g*^−1^ is equal to the identity element.

The order) of a group is the number of elements in the group. Let us assume *X* is a set of integers. Under the function composition operation, *Sym*(*X*) is a symmetry group, the set of all permutations over X. If the size of X is n, then the order of *Sym*(*X*) is *n*! Set) systems consist of a finite set *X* and its subsets, called blocks of the set. The set of permutations preserving the set system is used to build the automorphisms of the graph. An automorphism permutes the vertices of a graph; in other words, it maps a graph onto itself. This action is edge-vertex preserving. If (*u*,*v*) is an edge of the graph, *G* = (*V*,*E*), and *a* is a permutation of *V*, then

a(u,v)=(a(u),a(v)) (2)

A permutation *a* of *V* is an automorphism of the graph *G* = (*E*, *V*), if *a*(*u*, *v*) is an element of *E*, if (*u*, *v*) is an element of *E*.

**Fig 4:**
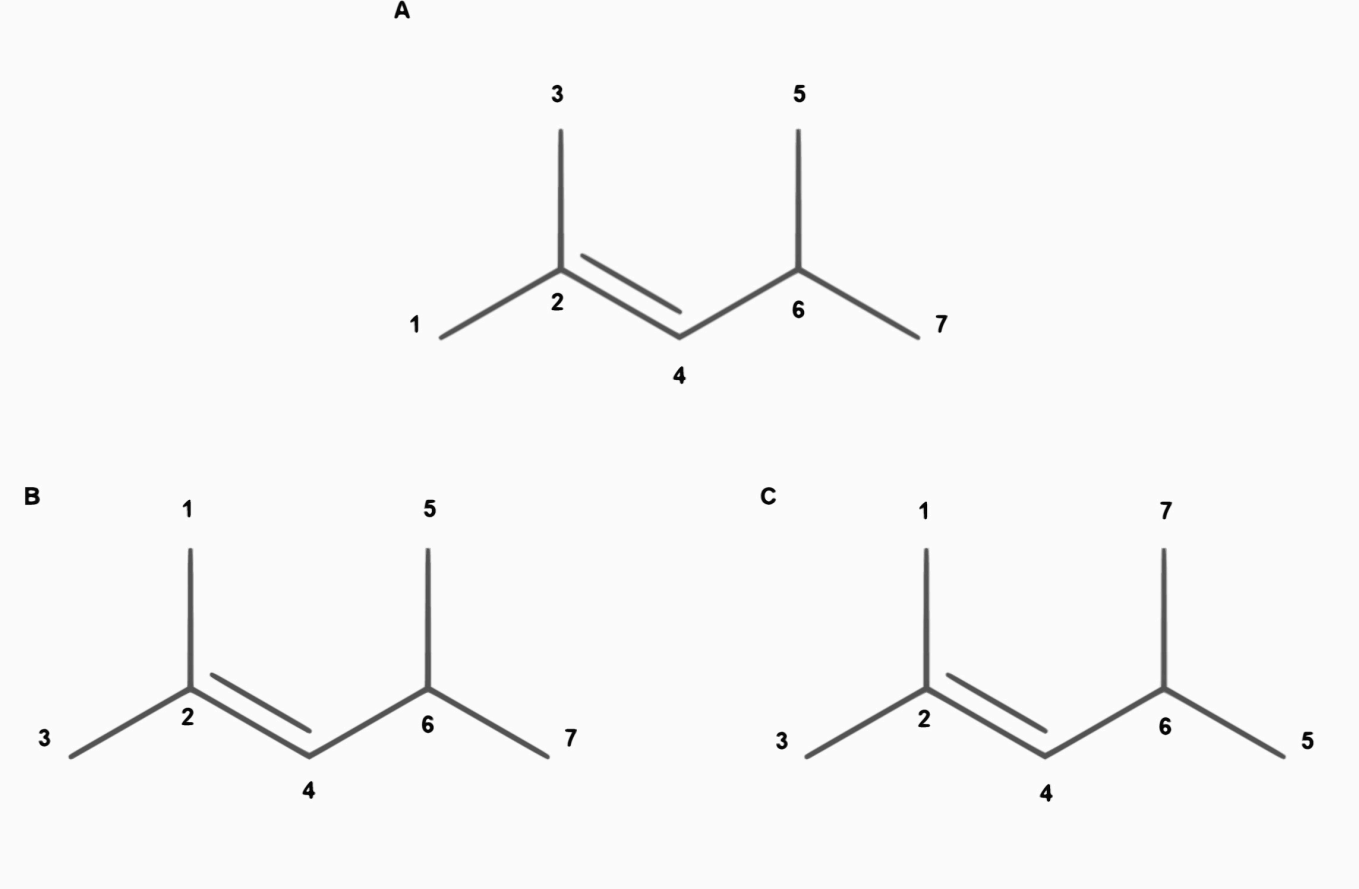
Molecular Symmetry. **(A)** The initial labelling of 2,4-Dimethyl-2-pentene. **(B)** and **(C)** are symmetries of the same molecule with different labels.

The automorphism group of a graph *G*, denoted *Aut*(*G*), is the set of all automorphisms on *V*. In molecular graphs, canonical labelling and molecular symmetry (Fig 4) detection are implementations of automorphism groups. Although there are well known canonical labelling methods in the field, such as InChI[45] and ALATIS[46], NAUTY is a commonly used software package for automorphism group calculations and canonical labelling.

## Conclusion

The structural identification of unknown molecules is an interdisciplinary field involving mathematicians, chemists and computer scientists; moreover, it has led to the creation of the field of mathematical chemistry and cheminformatics. The state-of-the-art methods comprise a variety of algorithms that can be classified into two groups; moreover, structure assembly has been the dominant approach in the field. Both assembly and reduction methods are incremental processes: all the intermediate structures are constructed based on previously generated structures, and duplicates are then excluded. The algorithms are generally breadth-first or depth-first search methods; and terminate once all the structures are saturated. The generation of too many intermediate structures and their storage make these algorithms inefficient. In the field, matrix generators have been attracting increasing interest from many scientists. According to the literature, there is still a lack of mathematical algorithms; more precisely, there is a lack of fast open-source structure generators.

### List of available structure generators

The available software packages and their links are listed below (Table 2).

**Table 2:** List of Available structure generators.

Name	Link
ASSEMBLE	http://www.upstream.ch/main.html?src=%2Findex.html
COCON	http://cocon.nmr.de
DENDRAL	http://www.softwarepreservation.org/projects/AI/DENDRAL/DENDRAL-CONGEN_GENOA.zip/view
LSD	http://eos.univ-reims.fr/LSD/index_ENG.html
MOLGEN	http://www.molgen.de/
MOLSIG	http://molsig.sourceforge.net
OMG	https://sourceforge.net/p/openmg/
PMG	https://sourceforge.net/projects/pmgcoordination/
SENECA	https://github.com/steinbeck/seneca
SMOG	http://ccl.net/cca/software/MS-DOS/SMOG/index.shtml

## Supporting information

S1 Text Version history of the text file. (XML)

S2 Text Peer reviews and response to reviews. (XML)
